# Deregulation of the cyclin-dependent kinase inhibitor p27 as a putative candidate for transformation in *Chlamydia trachomatis* infected mesenchymal stem cells

**DOI:** 10.3934/microbiol.2023009

**Published:** 2023-02-28

**Authors:** Mohammad A. Abu-Lubad, Wael Al-Zereini, Munir A. Al-Zeer

**Affiliations:** 1 Department of Medical Microbiology and Pathology, Faculty of Medicine, Mutah University, Al-Karak, Jordan; 2 Biological Sciences Department, Faculty of Science, Mutah University, Al-Karak, Jordan; 3 Department of Applied Biochemistry, Institute of Biotechnology, Technical University of Berlin, Berlin, Germany; 4 Department of Molecular Biology, Max Planck Institute for Infection Biology, Berlin, Germany

**Keywords:** p27, cell cycle arrest, cellular transformation, *Chlamydia trachomatis*, mesenchymal stem cells

## Abstract

**Purpose:**

Several pathological conditions might cause the degradation of the cyclin-dependent kinase inhibitor (CKI) p27 and cell cycle arrest at the G1 phase, including cancers and infections. *Chlamydia trachomatis* (Ctr), as an obligatory intracellular pathogen, has been found to alter the fate of the cell from different aspects. In this study, we aimed to investigate the effect of Ctr infection on the expression of the important cell cycle regularity protein p27 in mesenchymal stem cells (MSCs).

**Methods:**

Isolation of MSCs from healthy human fallopian tube was confirmed by detection of the stemness markers Sox2, Nanog and Oct4 and the surface markers CD44, CD73 and CD90 by Western blotting and fluorescence-activated cell sorting analysis. The expression of p27 was downregulated at the protein level upon Ctr D infection measured by Real-Time Quantitative Reverse Transcription PCR (qRT-PCR), IF and Western blotting. Recovery of p27 in Ctr D-infected MSCs was achieved by treatment with difluoromethylornithine (DFMO). Ctr D infected MSCs were able to produce colonies in anchorage-independent soft agar assay.

**Conclusion:**

Ctr D infection was able to downregulate the expression of the important cell cycle regulator protein p27, which will be considered a putative candidate for transformation in Ctr D infected MSCs.

## 1. Introduction

*Chlamydia trachomatis* (Ctr) is an obligate intracellular bacterium that causes a highly prevalent sexually transmitted disease [1]. It has a biphasic life cycle including the extracellular nonreplicating infectious elementary bodies (EBs) and the intracellular noninfectious replicating reticulate bodies (RBs) [2], which under unfavorable conditions progress into enlarged aberrant bodies (ABs) [3]. Different Ctr serovars are classified based on the major outer membrane protein (MOMP) structure into 15 serovars (A, B, Ba, C–K and L1–L3) [4]. They are grouped into trachoma biovar (serovars A–K) and the lymphogranuloma venereum (LGV) biovar (serovars L1–L3). The trachoma biovar is subdivided into endemic trachoma strains (serovars A–C) and oculogenital strains (serovars D–K) [4]. Although all serovars have >99.6% genetic identity, they differ in their disease outcomes and growth rates [4]. Ocular strains, genital strains and LGV strains grow at slow, intermediate and fast rates, respectively [5]. The genital tract infections of Ctr in women cause severe complications, including pelvic inflammatory disease (PID), leading to infertility, ectopic pregnancy and chronic pelvic pain [6]. The pathogenesis of chlamydia was studied using different infection cellular models, including primary cells, transformed cells and stem cells [7],[8]. Mesenchymal stem cells (MSCs) are one type of stem cell. MSCs have been isolated and characterized from the uterine cervix and the endometrium and are considered a target for Ctr infection [9]–[11]. Ctr infects the superficial endometrium epithelial layer, can reach the basal layer and infects the MSCs during menses, leaving the basal layer exposed to infection [8],[12]–[15]. A large body of studies has proved the association between Ctr infection and the alteration in the transcription of host genes in multiple cellular pathways [8],[16]–[21]. Some important pathways are the cell cycle associated oncogenes, tumor suppressors and the arginine metabolic pathways. We have shown previously that the proto-oncogene Myc is induced and stabilized in addition to the degradation of the tumor suppressor gene p53 through the activation of the PI3K pathway during chlamydial infection [18],[21]. The induction of Myc has also been found to induce the p27 degradation and eventually cellular proliferation [22]. In addition to that, Ctr infection induced the synthesis of polyamines through the upregulation of ornithine decarboxylase (ODC) and the downregulation of inducible nitric oxide synthase (iNOS) [8].

The cell cycle is a tightly regulated process that causes the cell to divide into two daughter cells. The regulation of cell cycle progression is governed by proteins of both the stimulatory cyclin/cyclin-dependent kinases (cyclin/CDKs) and the CDK inhibitory proteins (CKIs) [23],[24]. The cyclin/CDKs are negatively regulated by two groups of CKIs, including the inhibitor of CDK4 (INK4) proteins (p16^INK4A^, p15^INKB^, p18^INK4C^ and p19^INK4D^) and CDK-interacting protein/kinase inhibitory proteins (CIP/KIPs) (p21^CIP1^, p27^KIP1^ and p57^KIP2^) [25]. The regulatory protein p27 inhibits G1/S cell cycle progression by binding to and inhibiting the formation of the CDK6/cyclin D1, a complex that promotes the cell transition from the G1 phase to the S phase [26]. Low levels of p27 expression were detected in 60% of epithelial tissue in human carcinomas, contrary to its expression level in all normal epithelial tissues; the p27 deregulation in these cancers has been associated with accelerated proteolysis, sequestration and cytoplasmic mislocalization but not at the genetic level [27]. An important study showed that the cytoplasmic localization of p27 in mesenchymal stem cells may acquire oncogenic potential and drive subsequent metastasis [28]. Moreover, the expression of p27 was altered due to certain bacterial and viral infections; i*n vitro* and *in vivo* studies have highlighted the resistance of *Helicobacter pylori*-infected gastric cancer cells to apoptosis which was associated with low expression levels of p27 [29],[30], while murine herpesvirus 68 (MHV 68) infections might alter the fate of p27 [31]. Deregulation of certain metabolic pathways also alters the fate of p27. In our previous study, Ctr infection was found to alter the normal arginine metabolic pathway [8]. Arginine is an important amino acid utilized by two pathways that result in the production of polyamines and nitric oxide. The arginase enzyme catalyzes the conversion of arginine to ornithine and urea. L-ornithine can be further metabolized to polyamines via ODC and NO by nitric oxide synthase (NOS) [8],[32]. Polyamines have fundamental roles in cellular proliferation, transcriptional activation and cell survival [8]. We previously showed that Ctr D infection in MSCs upregulated the expression of ODC and downregulated the expression of iNOS [8]. In addition to chlamydia, many reports describe increased polyamine and ODC levels in various cancers [33],[34]. Cancer cells exhibited induction of ODC and polyamine synthesis, which are essential for cellular proliferation. The overexpression of ODC was found to induce tumorigenic transformation of rodent fibroblasts [35]. Neuroblastoma is an example in human tumor in which the inhibition of ODC by using difluoromethylornithine (DFMO) leads to the accumulation of p27, eventually causing cell cycle arrest [36].

To our knowledge, the effect of Ctr infection on the expression of p27 has not been investigated. Based on the studies that have demonstrated the association between ODC, polyamines and Myc, we aimed in this study to use Ctr-infected MSCs as a model to study the effect of infection on the expression of p27 protein and the MSCs' transformation.

## 2. Materials and methods

### 2.1. Isolation of MSCs from the fallopian tube

**Figure 1.**
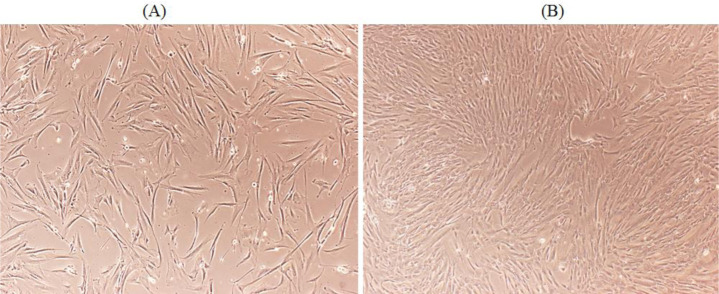
Microphotograph of isolated MSCs from hFTs. Cells were collected in DMEM/Hams F-12 and kept at 4 °C for processing within 24 h. MSCs in culture medium at 24 h (A) and 48 h (B).

MSCs were isolated from healthy fallopian tubes (hFTs) collected in DMEM/Hams F-12 medium (Invitrogen, Carlsbad, CA) and kept at 4 °C for processing within 24 h as described previously [15]. Briefly, hFTs were opened, washed twice in PBS (Gibco, Invitrogen, Carlsbad, CA) and incubated at 37 °C for 30 min in a 50 mL Falcon tube containing 5 mL of pure TrypLE™ Express (Invitrogen, Carlsbad, CA) with shaking. The supernatant was removed, washed once with 7 mL of advanced DMEM/Hams F12 in a 15 mL Falcon tube and centrifuged at 1000 rpm for 5 min at room temperature. The cells were then plated in advanced DMEM/Hams F12 (12 mL) supplemented with penicillin/streptomycin (100 µg/mL) in 75 cm^3^ polystyrene culture flasks and incubated at 37 °C in a humidified atmosphere containing 5.0% CO_2_ (Figure 1).

### 2.2. *C. trachomatis* propagation and infectivity titration assay

Ctr D (ATCC-VR885) propagation, preparation of EBs stock and estimation of the different multiplicities of infection (MOI)/mL were conducted using HeLa cells (ATCC-CCL2.1) grown in RPMI-1640 with incubation at 37 °C in a humidified atmosphere containing 5.0% CO_2_ as previously described [37].

### 2.3. Infection of MSCs with C. trachomatis and DFMO treatment

MSCs were cultured at a cell density of 1 × 10^5^ cell/well in 6-well plates and under standard cultivation conditions. Wells were divided into uninfected MSCs (NI), cells treated with 20 mM DFMO, infected cells for 72 h with Ctr D at MOI (1, 3, and 4) from the stock of 1.98 × 10^8^ IFU/mL and infected cells treated with 20 mM DFMO. To exclude the cytotoxic effect of 20 mM DFMO on MSCs' viability, the lactate dehydrogenase enzyme was measured in MDFMO-treated cells using the colorimetric lactate dehydrogenase (LDH) assay kit (Roche Diagnostics, Mannheim, Germany) following the manufacturer's instructions.

### 2.4. Effect of DFMO treatment on infectivity of C. trachomatis

The production of EBs in MSCs infected for 72 h with Ctr D (MOI 1, 3, and 4) and simultaneously treated with 20 mM DFMO was estimated using infectivity titration assays and compared to that in DFMO-untreated infected MSCs. Ctr D infected cells were removed by agitation with glass beads, mechanically lysed using glass beads and vigorously vortex-mixed for 3 min in 50 mL sterile tubes to release Ctr D. Then, 5 mL of the resulting bacterial homogenates, from either preparation, was used to infect and inoculated onto fresh (80% confluent) HeLa for 2 h. Post-infection, the inocula were removed, and the infected cells were washed 2–3 times with warm PBS. Finally, fresh pre-warmed medium was added, and the incubation was continued for an additional 48 h at 37 °C in a humidified atmosphere containing 5.0% CO_2_.

### 2.5. Detection of MSCs' stemness biomarker and p27 expression

#### 2.5.1. Flow cytometry

MSCs were cultivated at a cell density of 1.0 × 10^5^ cell/mL under standard cultivation conditions as described above. After 48 h of incubation, they were washed with warm PBS and trypsinized, and the cells were pelleted by centrifugation at 1200 rpm for 10 min. The cells were fixed in warm 4% paraformaldehyde (PFA), washed with PBS and stained with fluorescence-labeled antibodies (Abs) with incubation at 4 °C for 30 min. The used antibodies were specific for CD44 directly conjugated with fluorescein isothiocyanate (FITC) (1:100, Mouse, BD Pharmingen, USA), CD73- allophycocyanin (APC) (1:100, mouse, BioLegend, USA) and CD90- phycoerythrin (PE) (1:100, Mouse, BD Pharmingen, USA). Labeled cells were analyzed using a Fluorescence-activated cell sorting (FACS) instrument, and the results were depicted in histogram graphics.

#### 2.5.2. Immunofluorescence (IF) and confocal microscopy

Approximately 5 × 10^4^ MSCs were seeded on sterile coverslips in 12-well plates with incubation overnight at 37 °C in a humidified atmosphere containing 5.0% CO_2_. Cells were then statically infected with Ctr D at MOI of 1–4 for 72 h. Infected and uninfected cells were fixed with 4% PFA for 30 min. The fixed cells were washed 3 times with PBS, permeabilized in a blocking buffer with 0.03% (w/v) Triton 100X and finally blocked using the blocking buffer [0.3% bovine serum albumin (BSA) in PBS]; permeabilization and blocking were done for 30 min each at RT. Cells were incubated with the primary antibodies diluted in 0.3% BSA for 60 min at RT against p27 (1:100, mouse, clone: G173–524, BD Pharmingen), *C. trachomatis* lipopolysaccharide (1:5000, Clone: CF6J12, Abcam Cambridge, UK) and the white DNA staining DAPI-127 (Sigma-Aldrich, Germany). Primary antibody-labeled cells were washed with PBS and treated for 60 min at RT with secondary fluorescent anti-rabbit Cy3 labeled (Red) (Goat, 1:100, Dianova) and Anti-mouse Cy2 labeled (green) (Goat, 1:100, Dianova) Abs diluted in 1% BSA. β-actin was stained with the red color dye phalloidin. The preparation was washed 3 times with PBS for 5 min each at RT. All samples were mounted onto glass slides using Mowiol and examined by a Leica TCS-SP laser scanning confocal microscope (Leica Microsystems, Wetzlar, Germany).

### 2.6. Effect of DFMO treatment on C. trachomatis growth and p27 expression recovery in infected MSCs

#### 2.6.1. Western blotting

MSCs were seeded at a cell density of 1 × 10^5^ cell/well in 6-well plates with incubation overnight under standard conditions described above. They were infected with Ctr at MOI (1, 3 and 4) and cultivated for 72 h. Wells with uninfected cells were used as controls. For studying the effect of DFMO on the expression of p27, the infected cells were treated with 20 mM DFMO and compared with untreated infected cells. The cells were PBS washed and lysed with 200 µL of sodium dodecyl sulfate (SDS)-lysis buffer (3% 2-ME, 20% glycerin, 0.05% bromophenol blue, 3% SDS). After scraping using a rubber policeman, the lysate was collected in Eppendorf tubes and heated in a thermal block for 10 min at 96 °C. Equal amounts of protein were separated using SDS-PAGE, and immunoblotting detection of p27 expression was performed as described elsewhere [17].

#### 2.6.2. Quantitative reverse transcription PCR (RT-qPCR)

The total RNA was extracted from all cell preparations, uninfected and infected MSCs without and with DFMO (20 mM), using TRIzol QuantiTect SYBR Green PCR Kit (Power SYBR® Green 1-Step Kit, Applied Biosystems). The used oligonucleotides specific to p27 mRNA were the forward 5′-AAAAATCCGAGGTGCTTGG-3′ and the reverse 5′-ACAGCCCGAAGTGAAAAGAA-3′ primers [38]. The PCR reaction (25 µL final volume) involved 12.5 µL of SYBR Green master mix, 0.1 µL RNase inhibitor (stock: 20 U/mL), 0.2 µL reverse transcriptase, 8.4 µL purified RNA (10 ng/µL) and 1 µL from each primer. The conditions of amplification were a cycle at 48 °C for 30 min, followed by 40 cycles of 95 °C for 15 sec and 60 °C for 1 min. The cycling protocol was performed according to the manufacturer's instructions.

#### 2.6.3. Electron microscopy

The 6-well plates containing Ctr-infected MSCs treated and untreated with DFMO (20 mM) were taken at time intervals of 24 h, 48 h and 72 h, washed twice with cold PBS and fixed with 2.5% glutaraldehyde. Fixed cells were detached by a rubber policeman. The cells were post-fixed with 1% osmium tetroxide (OsO_4_) and then contrasted with tannic acid and uranyl acetate. The specimens were dehydrated in a graded ethanol series (50–100%) and embedded in agar 100. Ultrathin sections (70 nm) were produced, contrasted with lead citrate and examined with a Zeiss EM 10 scanning electron microscope and LEO 912AB transmission electron microscope (Carl Zeiss AG, Oberkochen, Germany).

### 2.7. Detection of MSCs' transformation by anchorage-independent soft-agar colony formation assay

Anchorage-independent growth was assessed in vitro using CytoSelect™ 96-Well Cell Transformation Assay, Soft Agar Colony Formation (Cell Biolabs, Inc., San Diego, CA) as per the manufacturer's guidelines. The main advantage of using this method is its accuracy in the quantification of colony formation compared to the classical techniques in manual counting of colony formation. Briefly, the wells were coated with a thin layer of a 1.2% agar solution, left to solidify. MSCs were grown in DMEM/Hams F-12 medium and infected with Ctr D (MOI 4). Two days post-infection, 7500 cells were suspended in 2 mL DMEM/Hams F-12 medium containing 0.6% agar, plated over the layer of the solidified agar and incubated at 37 °C in a humidified atmosphere containing 5.0% CO_2_ for 12 days. Wells were then photographed for cell growth on days 3, 6, 9 and 12. MSCs in the wells were lysed, and the DNA content was quantified after the addition of 90 µL of the CyQUANT working solution to each well. Wells were then incubated for 10 minutes at room temperature and were then read in a 96-well fluorometer using a 485/520 nm filter set.

## 3. Results

### 3.1. Isolation and characterization of MSCs

**Figure 2.**
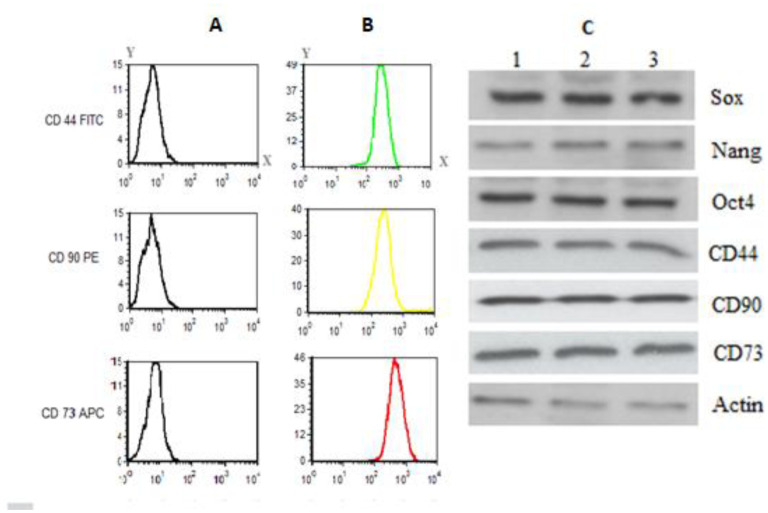
Representative histogram and Western blotting of the stemness markers expression in isolated MSCs. Cells isolated from fallopian tubes displayed stemness markers. The histograms in panel (B) show cells stained with anti-CD44 (FITS-green), anti CD90 (PE-yellow), and anti-CD73 (APC-red). The results were compared to unstained cells in panel (A). In panel (C), host cells were cultured and protein samples were collected at 72 h post-infection. Protein samples for the stemness markers Sox2, Nanog, Oct4, CD44, CD90, and CD73 were detected by Western blotting. β-actin was used as a loading control.

The isolated cells from the human fallopian tube were tested for their mesenchymal properties by detecting the stemness markers Sox2, Nanog, Oct4, CD44, CD73 and CD90 via Western blotting (Figure 2C) and CD44, CD73 and CD90 using FACS analysis (Figure 2A and B). FACS analysis showed high levels of CD44, CD73 and CD90 at laser excitation and emission wavelengths of 488 and 532 nm, respectively (Figure 2B). However, cells did not reveal expression of the epithelial markers E-cadherin and EpCAM (data not shown). All these detected features indicated the mesenchymal nature of the isolated cells.

### 3.2. The expression level of p27 in Ctr D infected MSCs

**Figure 3.**
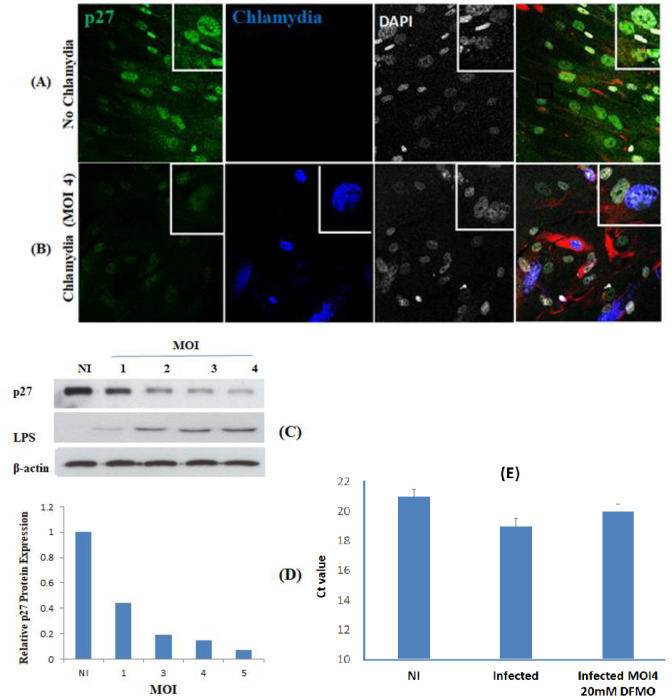
p27 expression in Ctr D infected and NI MSCs: MSCs were cultured for 72 h, fixed and stained by p27 (green), Ctr D LPS (Blue), and DNA DAPI-127 (white) immunofluorescent dyes. The signal of p27 was positive and localized in the nuclei of the NI cells (A), while it was very weak in Ctr D infected MSCs (B). Western blot for p27 in protein collected from MSCs collected from NI and infected cells with MOI (1-4) at 72 h post-infection along with the expression of LPS using the same MOIs, with β-actin used as a loading control (C). The p27 protein quantification in infected and NI cells (D). No difference in the RT-qPCR of p27 in NI, infected and infected 20 mM DFMO treated cells (E).

The effect of Ctr infection on the expression of p27 was investigated by immunofluorescence staining of MSCs, and signal level was detected by confocal microscope. In control NI cells, a strong p27 fluorescence signal was detected and localized in their nuclei. In contrast, the expression levels of p27 in infected cells were significantly downregulated compared to control samples (Figure 3A and B). Interestingly, results from Western blotting using MOIs from 1 to 4 showed an MOI-dependent dramatic and significant reduction in the relative intensity of the p27 protein band in the infected cells (Figure 3C) with a reduction in p27 protein expression (Figure 3D). The expression of the Ctr LPS showed increased signal with the increase in the MOI from 1 to 4 (Figure 3D), a quantitative measure confirming the immunohistochemical findings. Remarkably, the qRT-PCR showed comparable expression of mRNA in infected and NI cells (Figure 3E).

### 3.3. Effect of DFMO on C. trachomatis growth and recovery of p27 expression

**Figure 4.**
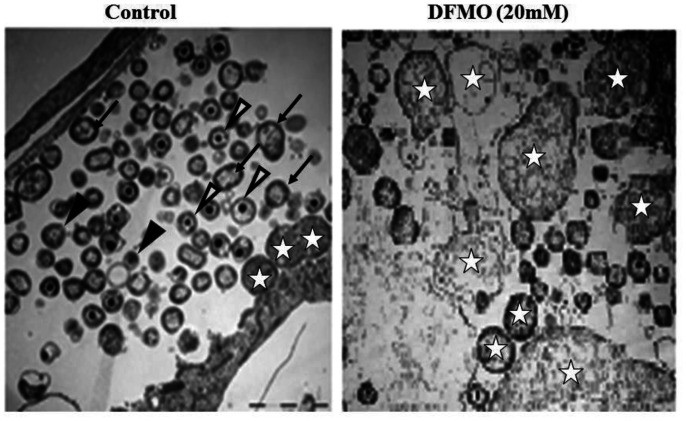
Effect of DFMO treatment on Ctr D growth. Transmission electron micrographs displaying ultrastructural features of Ctr D inclusions in MSCs in the presence of DFMO (20 mM). Untreated host cells show normal chlamydial inclusions with numerous reticulate (arrows), elementary (arrowheads) and intermediate (open arrowheads) bodies, with few aberrant bodies (stars). Wheresas, DFMO treated cells show morphologically abnormal chlamydial inclusions and the presence of numerous enlarged ABs, with few EBs.

**Figure 5.**
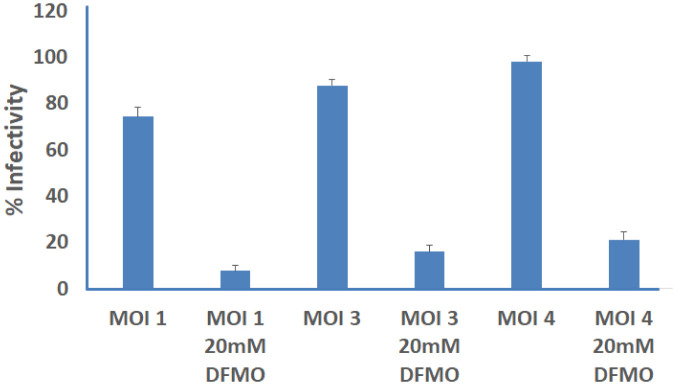
Effect of DFMO treatment on *C. trachomatis*infectivity. MSCs were infected for 72 h with Ctr D (MOI 1, 3, 4) and simultaneously treated with the 20 mM DFMO. The yield of Ctr D infectious progeny decreased considerably upon DFMO stimulation. Data were normalized to that in DFMO-untreated infected MSCs for each used MOI. The infectivity was expressed as a percentage ± standard deviation (SD) for three independent experiments.

The ability of DFMO in altering microbial normal growth and thereby restoring p27 expression lost due to infection was investigated in Ctr D-infected MSCs and compared with that in untreated infected cells. As shown in Figure 4, the electron microphotographs of infected MSCs at different time intervals showed normal growth and development of Ctr D. Meanwhile, treatment of infected MSCs with DFMO altered the normal development of Ctr D into aberrant bodies, which proved the negative effect of DFMO on the Ctr D growth. Moreover, correlated with the growth alteration effect of DFMO on the normal Ctr D growth and replication, a significant reduction in the infectivity of Ctr D using different MOI was noticed in those released from DFMO-treated MSCs compared with untreated cells (Figure 5). Figure 7 shows the relation between ODC, DFMO and p27 induction, as shown previously [8].

**Figure 6.**
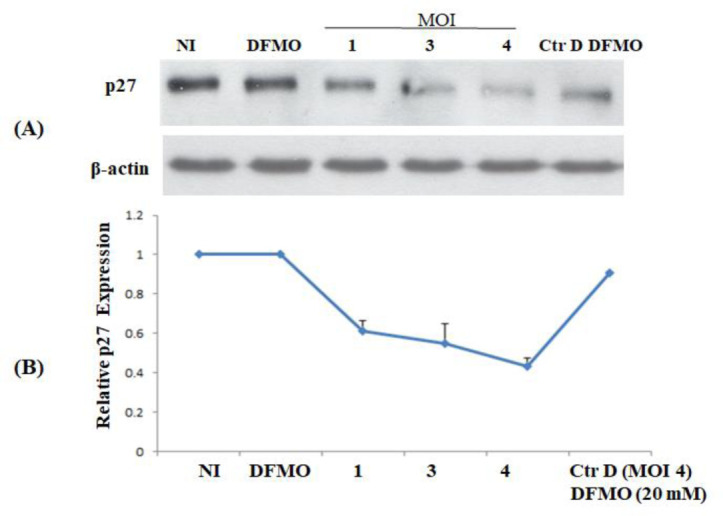
p27 expression in NI, DFMO treated, infected with MOI (1, 3, 4), and DFMO treated infected MSCs. Western blot of protein samples collected from MSCs under different conditions at 72 h post-infection with detection of p27 (A), with β-actin used as a loading control. p27 protein quantification in infected and NI cells with/without DFMO treatment (B).

Western blotting of total protein from NI, DFMO (20 mM) treated, infected at MOI (1, 3 and 4), and infected MSCs treated with DFMO (Figure 6A) revealed that p27 level decreased significantly in response to Ctr D infection and was restored upon treatment of infected cells with DFMO. p27 level was reduced by 60% at MOI of 4 in Ctr D infected MSCs, and DFMO treatment restored significantly the p27 expression to a level comparable to that in NI MSCs (Figure 6B).

**Figure 7.**
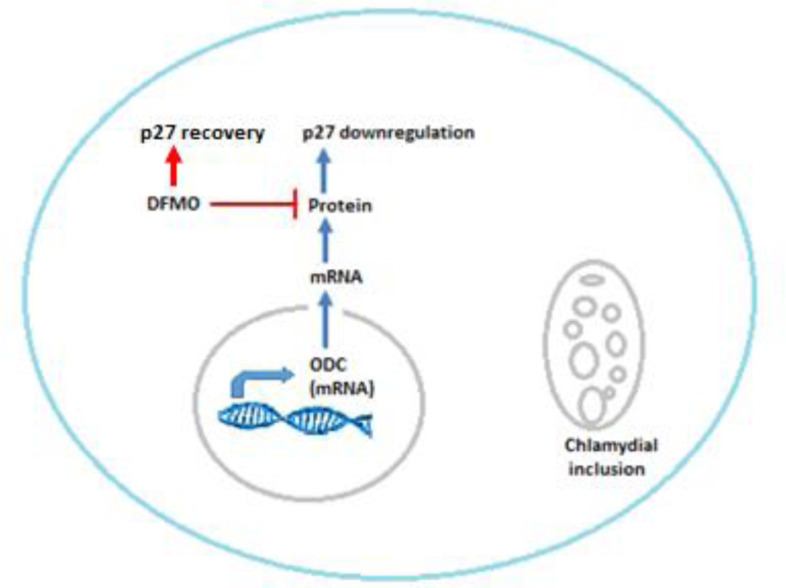
The relation between ODC, DFMO and p27 induction. ODC is induced after Ctr D infection, which eventually causes upregulation of polyamine synthetic pathway and p27 downregulation. The inhibition of ODC by DFMO recovered the expression of p27.

### 3.4. Quantitative measurement of MSCs' transformation by anchorage-independent soft-agar assay

**Figure 8.**
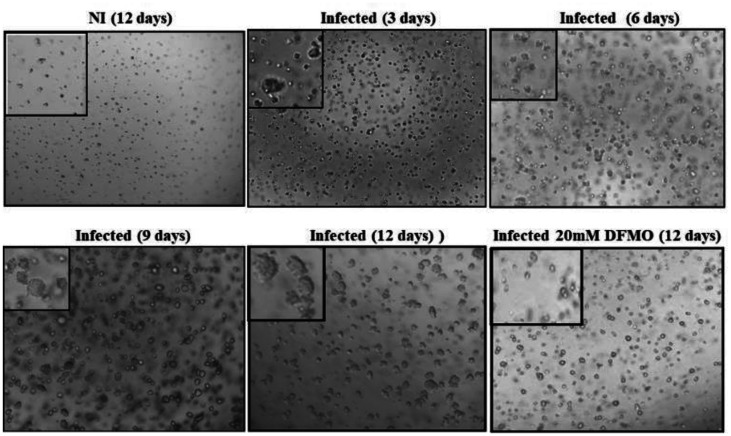
Colonies formed by Ctr D-infected MSCs in soft agar. MSCs were noninfected, infected with Ctr D (MOI 4) and infected treated with 20 mM DFMO. Two days post-infection, cells were seeded in soft agar for 12 days and observed for colony formation under a microscope (magnification 100× and 200×).

**Figure 9.**
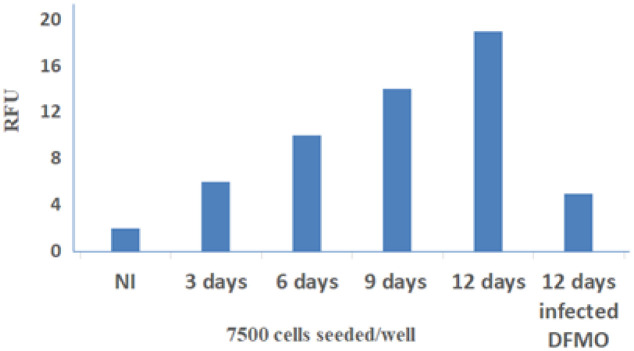
Quantitative determination of DNA content in colonies formed in anchorage-independent soft agar assay. Ctr D-infected MSCs were cultured for 12 days on soft agar. Cells were lysed, and DNA content was determined fluorometrically using the CyQUANT^®^ GR dye.

The effect of Ctr D infection on the transformation of MSCs was deduced by soft-agar growth assay. Cells were cultured for 12 days by observing the size of formed colonies in the wells under an inverted light microscope with colonies' photographing (Figure 8). The DNA content as an indication of the growth rate and size of Crt D-infected MSC colonies was determined in each well using relative fluorescence units (RFU) (Figure 9). The growth rate and the sizes of the colonies in Ctr D infected MSCs were significantly greater than those of the control uninfected and infected DFMO treated cells.

## 4. Discussion and conclusion

The association between Ctr infections and the increased risk of transformation and malignancy in host cells is still a controversial issue [39]. Some of the prevalent studies showed discrepancy regarding the risk of Ctr infection and cervical cancer [40]–[43]; it was confirmed that Ctr infection increased the risk of cervical and endometrial carcinomas through the induction of free radicals, cytokines and DNA damage [44],[45]. Intriguingly, the link between Ctr infection and transformation has been examined in several studies over the past decade. A study reported a link between Ctr and ovarian cancer (42); 9% of ovarian cancer has been found in women with a history of PID, though others showed that there was no connection between Ctr and ovarian cancer [46]. Moreover, in vivo studies on Ctr infection in mice showed significantly increased cervical cell proliferation and dysplasia [47]. Nevertheless, the mechanism by which Ctr might induce cellular transformation and tumorigenesis has yet to be elucidated [48]–[51]. As cellular transformation and malignancy entailed a loss in regulations of both tumor suppressor genes and proto-oncogenes, herein, the role of p27 as a putative tumor suppressor gene candidate for transformation in Ctr infected MSCs was addressed. The MSCs were used as a model for study since they are susceptible to Ctr infections during menstruation and surgical intervention; the isolation of MSCs from hFTs was confirmed by detecting the expression of different stemness and surface markers.

Remarkably, some pathogenic bacteria like *Chlamydia* species ensure their survival and growth by altering the cell cycle in infected host cells [52]; they enhance or suppress the normal cell cycle proliferation. *C. pneumoniae* infection of human endothelial cells induces proliferation of smooth muscle cells via an endothelial cell-derived soluble factor (s) [53]. On the contrary, Ctr L2 infection in HeLa and CHO-K1 cells reduced the rate of cellular proliferation through the reduction in the levels of cdk1 and cyclin B1 truncation [19], which was the case also with *H. pylori* infection in a gastric adenocarcinoma cell line [54]. Furthermore, *Escherichia coli* caused cell cycle arrest in HeLa cells through the inactivation of Cdk1 by phosphorylating the conserved tyrosine residue [55]. Human papillomavirus type 16 induced G2 arrest by cytoplasmic retention of active Cdk1/cyclin B1 and G2 arrest, while both human immunodeficiency virus type 1 and reovirus cause G2 arrest by inhibiting p34 (cdc2) activity [56]–[58]. In this study, we have chosen Ctr serovar D because it is well known that serovars D-K infect genital tract epithelial cells, resulting in pelvic inflammatory disease. In our study we used MSCs isolated from the fallopian tube, so we thought of using the D serovar. Other chlamydial strains infect different cells types. For example, Ctr serovars A-C infect conjunctival epithelial cells, and untreated infection can lead to blindness, while serovars L1–L3 infect epithelial cells and macrophages, causing an invasive infection [4]. In addition to that, in our previous study, DFMO showed almost equal effects on the growth of Ctr D and L2 serovars, so we chose Ctr D in this study [8].

Among the CKIs is p27^KIP1^, which is expressed at high nuclear levels in most normal epithelial tissues, and a loss or decrease in its level (i.e., deregulation) is commonly noticed in several cancer types with poor cancer prognosis [59]. Its role as a tumor suppressor protein is supported by the fact that inadequate levels of p27 in the G1 phase permit cells to transition from G1 to the S phase [60],[61], and mice lacking p27 suffer from multiorgan hyperplasia [62]. In cancers, different mechanisms of p27 downregulation are evident, including increasing of its proteolysis and the oncogenic overexpression of miRNAs that impair p27 translation [63],[64]; the regulation occurs at the transcriptional, translational and post-translational levels [65]. Herein, we revealed that Ctr D infection significantly downregulated the expression of p27KIP1 compared to the NI cells. The IF showed nuclear localization of p27 more evident in noninfected MSCs (NI) compared to Ctr-infected ones. The mRNA expression of p27 was comparable by RT-qPCR in both groups, though Western blotting indicated an altered expression of p27 in infected MSCs and highlighted a possible post-translational level regulation mechanism that requires further investigations. In similar studies, *H. pylori* infection caused a downregulation in p27 with mislocalized to the cytoplasm in gastric cancer [66] and decreases expression of p27 protein in H3S AGS gastric cells with a reduction in p27 mRNA; deregulation by ubiquitin independent proteasome-dependent pathway [66]. MHV 68 infections induced p27 degradation through its phosphorylation at threonine 187 (Thr^187^); phosphothreonine 187-p27^KIP1^ leads to polyubiquitination of p27 and subsequent proteolytic degradation by the Skp2/Cks1 ubiquitin-ligase complex [31],[67]. It is worth noting that regulation of p27 involves its sequestering in the cytoplasm by preventing the nuclear import through Thr-157 and Thr-198 phosphorylation [68] or by preventing nuclear export via Ser-10 phosphorylation and subsequent proteolysis by the Skp2 complex [69],[70], stabilizing p27 via phosphorylating it at Thr-197 [71] or Thr-198 and Ser-10 residues [72]. However, phosphorylation of p27 at the tyrosine residues -74, -88 and -89 reduces its inhibitory activity towards the Cdk4/6-cyclin D complex [71]. Decreased level of p27KIP1 will lead to sequestering the CIP/KIP inhibitors into CDK4/6-cyclin D and away from CDK2-cyclin E/A complex; thus, p27 will be a substrate to Cdk2-cyclin E/A rather than inhibitor, leading to p27 phosphorylation and degradation with activation of CDK2-cyclin E/A and cell progression from G1 to S phase [73].

In the present study, the influence of adding DFMO on the regulation of p27 in infected MSCs was evaluated. In a previous study, we confirmed the ability of DFMO as an irreversible inhibitor of ODC to alter *C. trachomatis* normal growth [8]. Herein, treatment of Ctr-infected MSCs with DFMO induced the formation of ABs and reduced the number of mature Ebs and intermediate bodies (IBs) Such alteration of bacterial growth was associated with recovery in the p27 content, which highlights the potential role of Ctr in downregulating p27 and promoting the host survival to facilitate bacterial growth and multiplication. The inhibition of ODC, a rate-limiting enzyme in polyamine synthesis, induced polyamine depletion; polyamines are considered among the important nitrogen sources for bacterial growth, especially those that thrive in nitrogen-limiting environments including human cells (e.g., intracellularly) [74]. Intriguingly, it was documented that DFMO treatments caused Ser-10 and Thr-198 phosphorylation in p27^Kip1^ and led to p27^Kip1^ accumulation in the cytoplasm, stabilizing the protein and decreasing its potential degradation [72].

The downregulation of p27 with Ctr D-infected MSCs indicated the anti-apoptotic effect of such infection on the host cells and the potential induction of cellular transformation. Therefore, an investigation on the ability of Ctr D to induce MSCs' transformation was performed. It was observed that Ctr D infection in MSCs was able to induce colony formation in soft agar, unlike with NI cells. Such finding was in line with the reported ability of Ctr L2 and *C. muridarum* Nigg strain infection to induce anchorage-independent growth in the 3T3 cellular transformation model and to alter the expression of the tumor suppressor gene p53 and the proto-oncogenes c-myc [18],[21],[75]. Furthermore, the human cytomegalovirus strain DB was able to inactivate retinoblastoma and p53 protein while activating oncogenic pathways with upregulation of cyclin D1 in infected mammary epithelial cells; the cells were able to form colonies in soft agar anchorage-independent growth assay [76]. Altogether, Ctr D infection caused downregulation in the p27 protein levels that promote host cell survival.

At this stage, the mechanism of p27 is still not clear. A study showed that the degradation of eleven proteins was due to enzymatic activity in the cell lysate and not associated with the chlamydia protease-like activity factor (CPAF) [77]. In this study, the downregulation was detected by immunofluorescence before exposing cells to cell lysis, indicating that the downregulation of p27 is not due to cell lysate enzymatic activity artifact, which was observed for other host factors as published before [77].

The p27 content was recovered by treating Ctr D-infected cells with DFMO by altering the growth of Ctr D and hence altering its anti-apoptotic effect. Moreover, it is postulated that p27 downregulation has a transformation potential in infected cells contributing to uncontrolled cellular proliferation. Therefore, these data support the hypothesis that Ctr D infection independently induced transformation and malignancy, and p27, as a tumor suppressor gene, is a new candidate for cellular transformation in Ctr D-infected MSCs.
